# Capitated versus fee-for-service reimbursement and quality of care for chronic disease: a US cross-sectional analysis

**DOI:** 10.1186/s12913-021-07313-3

**Published:** 2022-01-04

**Authors:** Sri Lekha Tummalapalli, Michelle M. Estrella, Deanna P. Jannat-Khah, Salomeh Keyhani, Said Ibrahim

**Affiliations:** 1 Division of Healthcare Delivery Science & Innovation, Department of Population Health Sciences, Weill Cornell Medicine, 402 East 67th Street, New York, NY 10065 USA; 2 Division of Nephrology & Hypertension, Department of Medicine, Weill Cornell Medicine, New York, NY USA; 3 Kidney Health Research Collaborative, Department of Medicine, San Francisco Veterans Affairs Medical Center and University of California, San Francisco, CA USA; 4 Division of Nephrology, Department of Medicine, San Francisco Veterans Affairs Medical Center, San Francisco, CA USA; 5 Department of Medicine, Weill Cornell Medicine, New York, NY USA; 6 Division of Rheumatology, Hospital for Special Surgery, New York, NY USA; 7 Division of General Internal Medicine, San Francisco Veterans Affairs Medical Center, San Francisco, CA USA

**Keywords:** Capitation, Fee-for-service, Physician reimbursement, Health services research, Chronic disease, Hypertension, Diabetes, Chronic kidney disease

## Abstract

**Background:**

Upcoming alternative payment models Primary Care First (PCF) and Kidney Care Choices (KCC) incorporate capitated payments for chronic disease management. Prior research on the effect of capitated payments on chronic disease management has shown mixed results. We assessed the patient, physician, and practice characteristics of practices with capitation as the majority of revenue, and evaluated the association of capitated reimbursement with quality of chronic disease care.

**Methods:**

We performed a cross-sectional analysis of visits in the United States’ National Ambulatory Medical Care Survey (NAMCS) for patients with hypertension, diabetes, or chronic kidney disease (CKD). Our predictor was practice reimbursement type, classified as 1) majority capitation, 2) majority FFS, or 3) other reimbursement mix. Outcomes were quality indicators of hypertension control, diabetes control, angiotensin-converting enzyme inhibitor or angiotensin receptor blocker (ACEi/ARB) use, and statin use.

**Results:**

About 9% of visits were to practices with majority capitation revenue. Capitated practices, compared with FFS and other practices, had lower visit frequency (3.7 vs. 5.2 vs. 5.2, *p* = 0.006), were more likely to be located in the West Census Region (55% vs. 18% vs. 17%, *p* < 0.001), less likely to be solo practice (21% vs. 37% vs. 35%, *p* = 0.005), more likely to be owned by an insurance company, health plan or HMO (24% vs. 13% vs. 13%, *p* = 0.033), and more likely to have private insurance (43% vs. 25% vs. 19%, *p* = 0.004) and managed care payments (69% vs. 23% vs. 26%, *p* < 0.001) as the majority of revenue. The prevalence of controlled hypertension, controlled diabetes, ACEi/ARB use, and statin use was suboptimal across practice reimbursement types. Capitated reimbursement was not associated with differences in hypertension, diabetes, or CKD quality indicators, in multivariable models adjusting for patient, physician, and practice characteristics.

**Conclusions:**

Practices with majority capitation revenue differed substantially from FFS and other practices in patient, physician, and practice characteristics, but were not associated with consistent quality differences. Our findings establish baseline estimates of chronic disease quality of care performance by practice reimbursement composition, informing chronic disease care delivery within upcoming payment models.

**Supplementary Information:**

The online version contains supplementary material available at 10.1186/s12913-021-07313-3.

## Background

Chronic diseases, including hypertension, diabetes, and chronic kidney disease (CKD), cause a tremendous disease burden and increase the risk of cardiovascular events, kidney failure, and mortality [1, 2]. Quality of care in chronic disease management remains suboptimal, with less than half of individuals receiving evidence-based therapies and achieving disease control [3, 4]. For example, analyses of the National Health and Nutrition Examination Survey showed that only 43.7% of participants with hypertension had controlled blood pressure < 140/90 mmHg, and only 50.5% of participants with diabetes had controlled Hemoglobin A1c (HbA1c) values < 7% [5, 6]. Emerging alternative payment models, which incorporate incentives for high-quality, cost-efficient care, aim to address quality of care gaps and rising expenditures in chronic disease management.

Although visit-based, fee-for-service (FFS) driven care remains the dominant reimbursement mechanism for outpatient visits in the US [7], capitation, which reimburses a set amount per patient per unit time, may be a promising alternative payment mechanism to FFS care [8]. Capitated payments were first introduced in the 1980s to control costs [9], and are now making a resurgence as a method to increase flexibility in care delivery and emphasize outcomes rather than volume. Furthermore, in the context of the Coronavirus Disease 2019 (COVID-19) pandemic, capitated payments provide a consistent revenue stream, leaving physician practices less financially vulnerable to decreases in visit volumes [10]. Two voluntary payment models through the Center for Medicare & Medicaid Innovation (CMMI) feature capitated payments as a central component: Primary Care First (PCF) [11] and Kidney Care Choices (KCC) [12]. In addition to a flat FFS primary care visit fee, PCF provides a capitated per beneficiary per month payment, tiered according to the average level of comorbidities in the practice. KCC provides a capitated payment quarterly to nephrology practices for aligned beneficiaries with CKD Stages 4 and 5.

The anticipated impact of capitated payments on the quality of chronic disease care is unclear, because prior studies examining the effect of capitated payments have shown mixed results [13]. Practices with predominantly capitated payments are less incentivized toward in-person visits, which may result in fewer opportunities to provide care and thus lower quality care [14, 15]. On the other hand, capitated payments coupled with quality incentives provide built-in compensation for non-visit-based care delivery, including care coordination, panel management, telephone calls, patient messaging, and other population health strategies, which may drive quality of care improvements.

The current reimbursement patterns of visits for patients with hypertension, diabetes, or CKD are currently not well understood. Therefore, using a national dataset of visits to office-based physicians, we examined the patient, physician, and practice characteristics of practices with capitation as the majority of revenue. We then assessed the variation in quality of chronic disease care by practice reimbursement type. These results could inform how resources should be targeted to improve chronic disease care in the United States.

## Methods

### Study design and population

We performed a serial cross-sectional analysis of visits to office-based ambulatory care physicians using data from the National Ambulatory Medical Care Survey (NAMCS). NAMCS is a federally funded survey conducted by the National Center for Health Statistics within the Centers for Disease Control and Prevention. NAMCS samples visits to office-based physicians seeing ambulatory patients, including physicians with private offices rented within a hospital. NAMCS excludes physicians who are federally employed, those in military service, and those who treat patients only in institutional settings (nursing homes or hospitals). NAMCS utilizes a stratified two-stage probability sample which is fully described elsewhere [16]; first, physicians were sampled nationally, then a systematic random sample of visits was selected for each physician during an assigned week for inclusion in NAMCS. Field representatives from the U.S. Census Bureau interviewed physicians or practice representatives on physician and practice characteristics. Data on patient and visit characteristics were abstracted and recorded by field representatives from electronic or paper medical charts using a standardized data collection form, available on the Centers for Disease Control and Prevention website [16].

For our analysis, we included follow-up visits in NAMCS from 2012 to 2016 for adults (age ≥ 18) with hypertension, Type 2 diabetes, or recognized CKD. Hypertension was defined as having an ICD-9 or ICD-10 code consistent with hypertension, receipt of antihypertensive medications, or a physician-reported diagnosis of hypertension in the medical chart. Diabetes was defined as having an ICD-9 or ICD-10 code for Type 2 diabetes, receipt of antidiabetic medications, or a physician-reported diagnosis of Type 2 diabetes. CKD was determined based on an ICD-9 or ICD-10 code for CKD, or a physician-reported diagnosis in the medical chart of “chronic renal failure” (2012–2013) or “chronic kidney disease” (2014–2016). Visits to the following specialties were included: general/family practice, internal medicine, cardiology, and medical “other specialties” according to NAMCS classification. We did not include new patient visits (*n* = 4501) because chronic disease management would be less attributable to the physician for new patients. We did not include visits to obstetrics and gynecology, general surgery, orthopedic surgery, dermatology, urology, psychiatry, neurology, ophthalmology, and otolaryngology, as these visits were unlikely to be primarily for chronic disease care. Pregnant adults (*n* = 455) and patients with a diagnosis of end-stage kidney disease (*n* = 10) were excluded.

### Predictors and study covariates

Our predictor of interest was practice reimbursement composition, which was classified into three mutually exclusive categories: 1) majority (> 50%) capitated payments, 2) majority (> 50%) fee-for-service (FFS) revenue, or 3) other mix of fee-for-service, capitation, case rates (e.g. package pricing/episode of care), or other sources. Capitation reimbursement was assessed by asking “The following questions are about your practice revenue…Roughly, what percent of your patient care revenue comes from capitation?” FFS reimbursement was assessed by the question “Roughly, what percent of your patient care revenue comes from usual, customary, and reasonable fee-for-service?” Case rates was asked by the question “Roughly, what percent of your patient care revenue comes from case rates (e.g. package pricing/episode of care)?” and other sources was assessed by asking “Roughly, what percent of your patient care revenue comes from other sources?”

Study covariates included patient characteristics and physician/practice characteristics. We assessed the following patient characteristics: age, sex, race (non-Hispanic white, non-Hispanic Black, Hispanic, non-Hispanic other), comorbidities (cancer, cerebrovascular disease, chronic obstructive pulmonary disease, congestive heart failure, coronary artery disease, depression, and obesity), total of number of chronic conditions, and patient payor type (private insurance, Medicare, Medicaid, or other). Comorbidities were defined by physician-reported diagnoses in the medical chart, except for obesity, which was defined as a body mass index of 30 kg/m^2^ or greater. Physician/practice characteristics included practice location (United States Census Region, metropolitan statistical area), practice size (solo or group), physician specialty (primary care or medical specialty care), physician compensation type (share of billings, fixed salary, mix, or other), practice ownership (physician, medical/academic health center, or insurance company/health plan/health maintenance organization), physician employment status (full owner, part owner, employee or contractor), practice payor mix (majority Medicare, majority Medicaid, majority private insurance, majority patient payments or other), and managed care contract revenue.

### Outcomes

The primary study outcomes were quality indicators of hypertension, diabetes, and CKD care. The hypertension quality indicator was controlled hypertension with systolic blood pressure (BP) < 140 mmHg and diastolic BP < 90 mmHg, according to guidelines during the study period [17, 18]. Diabetes quality indicators were 1) controlled diabetes (HbA1c < 7%) among persons with diabetes [19]; 2) angiotensin-converting enzyme inhibitor or angiotensin receptor blocker (ACEi/ARB) use among those with hypertension and diabetes [20]; and 3) statin use in those with diabetes and aged 40–75 [21]. CKD quality indicators were 1) controlled hypertension (systolic BP < 130 mmHg and diastolic BP < 80 mmHg) among those with hypertension and CKD [17]; 2) controlled diabetes (Hemoglobin A1c [HbA1c] < 7%) among persons with diabetes and CKD [19]; 3) ACEi/ARB use in those with hypertension and CKD [20]; and 4) statin use if age ≥ 50 and CKD [22]. We performed sensitivity analyses varying the definition of controlled hypertension and defining controlled diabetes as an HbA1c < 8% [23]. Medications were coded in NAMCS using Lexicon Plus, a comprehensive database of all prescription and some nonprescription drug products available in the U.S. [24]. The NAMCS dataset was fully de-identified and therefore was not subject to Institutional Review Board review.

### Statistical analysis

We first compared patient and physician/practice characteristics for visits by patients with chronic disease by practice reimbursement type: 1) majority capitation, 2) majority FFS, and 3) other reimbursement mix. Differences in characteristics across practice reimbursement types were assessed using Wald tests to test the joint significance of the coefficients of a categorical variable being simultaneously equal to zero (Table 1). We additionally performed multivariable logistic regression to examine patient and physician/practice characteristics independently associated with having the majority of practice revenue from capitation (Supplemental Table 1).

**Table 1:** Characteristics of U.S. Office-based Visits for Patients with Chronic Disease by Practice Reimbursement Composition (*N* = 41,897)

	Majority Capitation (***N*** = 2316)	Majority FFS (***N*** = 33,569)	Other Reimbursement Mix (***N*** = 6012)	***p***-value
**Patient Characteristics**				
**Demographics**				
Age	66 [14]	65 [14]	64 [14]	0.056
Sex				
Male	45%	47%	44%	0.096
Female	55%	53%	56%	
Race/Ethnicity				
Non-Hispanic White	51%	74%	63%	< 0.001
Non-Hispanic Black	15%	12%	14%	
Hispanic	24%	9%	17%	
Non-Hispanic Other	9%	5%	6%	
**Comorbidities**				
Cancer	9%	9%	8%	0.546
Cerebrovascular Disease	4%	4%	3%	0.393
COPD	7%	8%	8%	0.745
Congestive Heart Failure	7%	5%	4%	0.017
Coronary Artery Disease	16%	15%	12%	0.075
Depression	12%	12%	11%	0.700
Obesity	43%	48%	45%	0.258
Total Number of Chronic Conditions	3.1 [1.7]	2.8 [1.7]	2.7 [1.6]	0.032
**Payor Type**				
Private Insurance	35%	40%	38%	0.034
Medicare	53%	51%	49%	
Medicaid	7%	6%	8%	
Other	4%	3%	5%	
**Visit Characteristics**				
Number of Times Seen in Past 12 Months	3.7 [4.8]	5.2 [4.0]	5.2 [5.4]	0.006
**Physician/Practice Characteristics**				
**United States Census Region^**				
Northeast	10%	20%	25%	< 0.001
Midwest	7%	23%	16%	
South	30%	39%	42%	
West	53%	18%	17%	
**Metropolitan Area**	93%	88%	91%	0.081
**Solo Practice**	21%	37%	35%	0.005
**Physician Specialty**				
Primary Care	77%	67%	71%	0.135
Medical Specialty Care	23%	33%	29%	
**Physician Compensation**				
Share of Billings	8%	23%	22%	< 0.001
Fixed Salary	58%	32%	37%	
Mix	27%	37%	30%	
Other	7%	8%	11%	
**Practice Ownership**				
Physician	66%	76%	79%	0.033
Medical/Academic Health Center	10%	12%	8%	
Insurance Company, Health Plan or HMO	24%	13%	13%	
**Employment Status**				
Full Owner	22%	39%	38%	0.004
Part Owner	28%	23%	24%	
Employee or Contractor	50%	38%	38%	
**Payor Mix**				
Majority Medicare	27%	27%	23%	0.488
Majority Medicaid	4%	2%	2%	0.298
Majority Private Insurance	43%	25%	19%	0.004
Majority Patient Payments or Other*	1%	1%	4%	0.041
**Majority Managed Care Contracts**	69%	23%	26%	< 0.001

Continuous variables listed as mean [standard deviation]. Categorical variables reported as percentages. Percentages may not add to 100% due to rounding. *P*-values are from Wald tests of unadjusted logistic and linear regressions to used to test the joint significance of the coefficients of a categorical variable (practice reimbursement composition) being simultaneously equal to zero

Comorbidities were based on physician-reported diagnosis, except for obesity, which was defined as a body mass index of 30 kg/m^2^ or greater. Hypertension and diabetes were defined using ICD-9 codes, use of medications for hypertension and diabetes, or physician-reported hypertension or diabetes

*COPD* chronic obstructive pulmonary disease, *HMO* Health Maintenance Organization

United States Census Regions^ are as follows: Northeast – Connecticut, Delaware, Maryland, Massachusetts, Maine, New Hampshire, New Jersey, New York, Pennsylvania, Rhode Island, Vermont, Virginia, West Virginia; Midwest – Illinois, Indiana, Iowa, Kansas, Michigan, Minnesota, Missouri, Nebraska, North Dakota, Ohio, South Dakota, Wisconsin; South – Alabama, Arkansas, Delaware, Florida, Georgia, Kentucky, Louisiana, Maryland, Mississippi, North Carolina, Oklahoma, South Carolina, Tennessee, Texas, Virginia, Washington, DC, West Virginia; West – Alaska, Arizona, California, Colorado, Hawaii, Idaho, Montana, Nevada, New Mexico, Oregon, Utah, Washington, Wyoming

*Other includes charity, research, CHAMPUS, and the VA

We then calculated unadjusted chronic disease quality of care indicator performance, according to numerator and denominator definitions presented in Table 2, stratified by practice reimbursement type. We used multivariable logistic regression to evaluate the cross-sectional association between practice reimbursement type and chronic disease quality indicators using two nested models. Model 1 adjusted for patient characteristics, including age, sex, race, comorbidities, total number of chronic conditions, and payor type in order to assess the association of reimbursement type with quality of care, after adjusting for differences in patient population. Model 2 additionally adjusted for physician/practice characteristics, including practice location, practice size, physician specialty, physician compensation, practice ownership, and physician employment status, to control for structural factors other than reimbursement type than may influence chronic disease care quality. We accounted for multiple comparisons using a Bonferroni correction, where a two-sided *p*-value of 0.003 was considered statistically significant for our analyses.

**Table 2:** Definitions of Quality of Care Indicators

Quality Indicator	Numerator (visits by adults with)	Denominator (visits by adults with)	Quality Indicator Source	Strength of Recommendation/Level of Evidence
**Hypertension**				
Controlled Hypertension	Systolic BP < 140 and Diastolic BP < 90 or Systolic BP < 130 and Diastolic BP < 80	Hypertension (physician-reported or ICD-9 code or receipt of antihypertensive)	JNC 7 [17] (2003) and JNC 8 [18] (2014)	E^a^
**Diabetes**				
Controlled Diabetes	HbA1c < 7% or HbA1c < 8%	Diabetes (physician-reported or ICD-9 code or receipt of anti-diabetic agent)	ADA [23] (2012)	B^b^
ACEi/ARB use	ACEi/ARB listed or dispensed during visit	1) Diabetes (physician-reported or ICD-9 code or receipt of anti-diabetic agent) and 2) Hypertension (physician-reported or ICD-9 code or receipt of antihypertensive)	ADA [23] (2012)	C^c^
Statin use	Statin listed or dispensed during visit	1) Diabetes (physician-reported or ICD-9 code or receipt of anti-diabetic agent) and 2) Age 40–75	ACC/AHA [21] (2013)	IA^d^
**Chronic Kidney Disease**				
Controlled Hypertension	Systolic BP < 130 and Diastolic BP < 80 or Systolic BP < 140 and Diastolic BP < 90	1) CKD (physician-reported or ICD-9 code) and 2) Hypertension (physician-reported or ICD-9 code or receipt of antihypertensive)	JNC 7 [17] (2003) and ACC/AHA [25] (2017)	E^a^
Controlled Diabetes	HbA1c < 7% or HbA1c < 8%	1) CKD (physician-reported or ICD-9 code) and 2) Diabetes (physician-reported or ICD-9 code or receipt of anti-diabetic agent)	KDOQI [19] (2012) ADA [23] (2012)	1A^e^
ACEi/ARB use	ACEi/ARB listed or dispensed during visit	1) CKD (physician-reported or ICD-9 code) and 2) Hypertension (physician-reported or ICD-9 code or receipt of antihypertensive)	KDIGO CKD [20] (2012)	1B^f^, 2D^g^
Statin use	Statin listed or dispensed during visit	1) CKD (physician-reported or ICD-9 code) and 2) Age ≥ 50	KDIGO Lipid [22] (2013)	1A^e^, 1B^f^

*CKD* chronic kidney disease, *ACEi* Angiotensin converting enzyme inhibitor, *ARB* angiotensin receptor blocker, *NSAID* nonsteroidal anti-inflammatory drug, *HbA1c* Hemoglobin A1c, *ICD* International Classification of Diseases, *JNC* Joint National Committee, *KDOQI* Kidney Disease Outcomes Quality Initiative, *ADA* American Diabetes Association, *KDIGO* Kidney Disease Improving Global Outcomes

^a^ E - Expert Opinion

^b^ B - Supportive evidence from well-conducted cohort or case-control studies

^c^ C - Supportive evidence from poorly controlled or uncontrolled studies or conflicting evidence with the weight of evidence supporting the recommendation

^d^ IA – Procedure/treatment should be performed/administered, data derived from multiple randomized clinical trials or meta-analyses

^e^ 1A - Level 1 “We recommend,” High quality of evidence

^f^ 1B - Level 1 “We recommend,” Moderate quality of evidence

^g^ 2D - Level 2 “We suggest,” Very low quality of evidence

All analyses incorporated patient visit weights and accounted for the ultimate cluster sampling in NAMCS, as described in NAMCS micro-data file documentation (Appendix 1). A total of 27% of visits for patients with hypertension, diabetes, or CKD had missing reimbursement information. To eliminate potential bias from complete case analysis, missing predictors and covariates were estimated using multiple imputation by chained equations using 50 imputations. Missingness of covariates was as follows: patient payor type (6%), physician compensation (5%), practice ownership (4%), physician employment status (3%), practice payor mix (14%), and managed care contract revenue (21%). Regression equations composed of all variables used in the fully adjusted models were created to impute missing values. We did not impute the outcomes of controlled hypertension or diabetes, as blood pressure and HbA1c laboratory values were thought to be missing not at random. We performed diagnostics using the Stata command midiagplots which showed good fitness of the generated data sets [26]. Data analyses were performed using Stata/IC, version 15.1 (StataCorp) and R version 4.0.2 statistical software, and all results presented are weighted estimates from the imputed dataset.

## Results

### Patient and practice characteristics

Between 2012 to 2016, there were 41,834 unweighted visits to 3766 unique physicians for patients with hypertension, diabetes, or CKD in our study population, representing 1,091,331,663 weighted visits. In weighted analyses, about 9% (95% confidence interval [CI] 6.3–11.1%) of visits were to practices with majority capitation revenue; 76% (72.9–78.5%) were to those with majority FFS revenue, and 16% (13.6–17.5%) to practices with another reimbursement mix. Patients in our study population visiting practices with majority capitated revenue were more likely to be Black or Hispanic, have congestive heart failure, and have Medicare as their primary payment source (Table 1). Age and sex did not statistically differ by practice reimbursement type. Patients visiting practices with majority capitated reimbursement were seen fewer times in the past 12 months (3.7 vs. 5.2 vs. 5.2, *p* = 0.006), compared with practices with majority FFS revenue or other reimbursement mix.

**Fig. 1:**
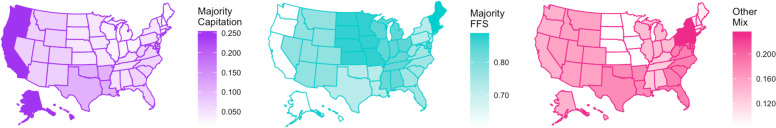
Variation in Practice Reimbursement Composition for Chronic Disease Visits by Census Division. Figure constructed by authors using R version 4.0.2 statistical software. Shading corresponds to the proportion of practices in each practice reimbursement type

There was significant regional variation in reimbursement type for chronic disease visits (Fig. 1). Compared with FFS practices and other reimbursement practices, majority capitated practices were more likely to be in the West Census Region (55% vs. 18% vs. 17%, *p* < 0.001). Capitated practices were also less likely to be solo practice than FFS and other reimbursement mix practices (21% vs. 37% vs. 35%, *p* = 0.005). Capitated practices were more likely to be paid with a fixed salary, rather than a share of billings (58% vs. 32% vs. 37%, *p* < 0.001), and more likely to be an employee or contractor (50% vs. 38% vs. 38%, *p* = 0.004), compared with FFS and other practices*.* Capitated practices were more likely to be owned by an insurance company, health plan or HMO, compared with FFS and other practices (24% vs. 13% vs. 13%, *p* = 0.033). Capitated practices more likely to have a majority of their practice’s patient care revenue coming from private insurance (43% vs. 25% vs. 19%, *p* = 0.004) and managed care payments (69% vs. 23% vs. 26%, *p* < 0.001), compared with FFS and other practices. Patient and physician/practice characteristics did not otherwise differ across practice reimbursement types.

### Hypertension quality indicators

The prevalence of controlled hypertension (BP < 140/90 mmHg) was similar across capitated, FFS, and other reimbursement types in unadjusted analyses (71% vs. 74% vs. 75%, Table 3). The odds of controlled hypertension in visits to capitated practices did not differ significantly from those to FFS practices in multivariable analyses adjusted for patient characteristics. In the fully adjusted model, the odds of controlled hypertension in capitated practices was not statistically significantly different than for FFS practices, adjusting for both patient and physician/practice characteristics.

**Table 3:** The Association of Reimbursement Composition and Hypertension and Diabetes Quality Indicators

Reimbursement Composition	Controlled Hypertension (BP < 140/90) in Patients with Hypertension^**a**^ % or aOR (95% CI) (***N*** = 36,540)	***p***-value	Controlled Diabetes (HbA1c < 7%) in Patients with Diabetes^**b**^ % or aOR (95% CI) (***N*** = 6016)	***p***-value	ACEi/ARB Use in Patients with Diabetes and Hypertension % or aOR (95% CI) (***N*** = 11,983)	***p***-value	Statin Use in Patients Age 40–75 with Diabetes % or aOR (95% CI) (***N*** = 10,985)	***p***-value
**Unadjusted Prevalence**								
Majority Capitation	71%	0.388	57%	0.586	54%	0.341	37%	0.431
Majority FFS	74%	*(ref.)*	59%	*(ref.)*	50%	*(ref.)*	40%	*(ref.)*
Other Revenue Mix	75%	0.499	63%	0.268	46%	0.131	32%	0.004
**Model 1**								
Majority Capitation	0.84 (0.60–1.18)	0.313	0.98 (0.65–1.49)	0.932	1.09 (0.81–1.46)	0.589	0.80 (0.54–1.20)	0.282
Majority FFS *(ref.)*	1	–	1	–	1	–	1	–
Other Revenue Mix	1.09 (0.89–1.33)	0.399	1.42 (1.01–2.00)	0.044	0.80 (0.60–1.07)	0.136	0.68 (0.50–0.90)	0.009
**Model 2**								
Majority Capitation	0.86 (0.65–1.15)	0.318	0.90 (0.59–1.38)	0.640	1.03 (0.74–1.44)	0.864	0.86 (0.59–1.25)	0.425
Majority FFS *(ref.)*	1	–	1	–	1	–	1	–
Other Revenue Mix	1.10 (0.90–1.34)	0.355	1.36 (0.98–1.90)	0.069	0.83 (0.61–1.11)	0.210	0.69 (0.52–0.93)	0.014

*CKD* chronic kidney disease, *BP* blood pressure, *aOR* adjusted odds ratio, *CI* confidence interval, *HbA1c* Hemoglobin A1c, *ACEi* Angiotensin converting enzyme inhibitor, *ARB* angiotensin receptor blocker, *NSAID* nonsteroidal anti-inflammatory drug

Differences in the unadjusted prevalence of quality indicators across reimbursement types were assessed using unadjusted logistic regression

Model 1 – adjusted for patient characteristics: age, sex, race, comorbidities, total number of chronic conditions, and patient payor type

Model 2 – adjusted for Model 1 + physician/practice characteristics: United States Census Region, metropolitan statistical area, solo practice, physician specialty, physician compensation, practice ownership, and physician employment status

^a^Hypertension and nonmissing blood pressure reading

^b^Diabetes and nonmissing HbA1c data

### Diabetes quality indicators

The prevalence of controlled diabetes (HbA1c < 7%) was similar across capitated, FFS, and other practices in unadjusted analyses (57% vs. 59% vs. 63%, Table 3). ACEi/ARB use among those with diabetes and hypertension was suboptimal across reimbursement types (54% vs. 50% vs. 46%). Recommended statin use was also low across capitated, FFS, and other practices (37% vs. 40% vs. 32%).

In either multivariable model, there were no statistically significant associations between reimbursement type and controlled diabetes or ACEi/ARB use, among visits for diabetes. In analyses adjusted for patient characteristics, other revenue mix practices had lower odds of statin use among visits for diabetes, compared with FFS practices (adjusted odds ratio [aOR] 0.68, 95% CI [0.50–0.90]). When additionally adjusted for physician/practice characteristics, other revenue mix practices remained associated with lower statin use among visits for diabetes (aOR 0.69, 95% CI [0.52–0.93]), but this did not reach statistical significance when accounting for multiple comparisons.

### CKD quality indicators

Patient visits for CKD to majority capitated practices were less likely to have controlled hypertension (BP < 130/80 mmHg) in unadjusted analyses, compared with FFS or other practices (43% vs. 56% vs. 57%, Table 4). Across practice reimbursement types, the prevalence of controlled diabetes (HbA1c < 7%) among patients with CKD was higher but not statistically different in capitated practices (78% vs. 59% vs. 61%). Less than half of patients with CKD were prescribed ACEi/ARBs and less than one-third were prescribed statins across reimbursement types. Prevalence of ACEi/ARB use among CKD visits was similar across capitated, FFS, and other reimbursement types (45% vs. 45% vs. 40%), and statin use was lower but not statistically different in capitated practices (21% vs. 35% vs. 31%).

**Table 4:** The Association of Reimbursement Composition and CKD Quality Indicators

Reimbursement Composition	Controlled Hypertension (BP < 130/80) in Patients with CKD and Hypertension^**a**^ % or aOR (95% CI) (***N*** = 3028)	***p***-value	Controlled Diabetes (HbA1c < 7%) in Patients with CKD and Diabetes^**b**^ % or aOR (95% CI) (***N*** = 579)	***p***-value	ACEi/ARB Use in Patients with CKD and Hypertension % or aOR (95% CI) (***N*** = 3110)	***p***-value	Statin Use in Patients Age ≥ 50 with CKD % or aOR (95% CI) (***N*** = 3381)	***p***-value
**Unadjusted Prevalence**								
Majority Capitation	43%	0.040	78%	0.055	45%	0.994	21%	0.075
Majority FFS	56%	*(ref.)*	59%	*(ref.)*	45%	*(ref.)*	35%	*(ref.)*
Other Revenue Mix	57%	0.829	61%	0.758	40%	0.417	31%	0.557
**Model 1**								
Majority Capitation	0.61 (0.34–1.08)	0.091	2.57 (0.93–7.10)	0.068	1.02 (0.67–1.54)	0.924	0.55 (0.21–1.42)	0.215
Majority FFS *(ref.)*	1	–	1	–	1	–	1	–
Other Revenue Mix	1.12 (0.66–1.90)	0.667	1.35 (0.67–2.74)	0.401	0.88 (0.50–1.57)	0.671	0.99 (0.58–1.69)	0.957
**Model 2**								
Majority Capitation	0.65 (0.38–1.11)	0.113	2.87 (1.02–8.06)	0.046	0.77 (0.45–1.32)	0.339	0.75 (0.40–1.38)	0.350
Majority FFS *(ref.)*	1	–	1	–	1	–	1	–
Other Revenue Mix	1.07 (0.62–1.87)	0.804	1.49 (0.74–3.00)	0.260	0.91 (0.50–1.63)	0.744	0.98 (0.59–1.64)	0.948

*CKD* chronic kidney disease, *BP* blood pressure, *aOR* adjusted odds ratio, *CI* confidence interval, *HbA1c* Hemoglobin A1c, *ACEi* Angiotensin converting enzyme inhibitor, *ARB* angiotensin receptor blocker, *NSAID* nonsteroidal anti-inflammatory drug

Differences in the unadjusted prevalence of quality indicators across reimbursement types were assessed using unadjusted logistic regression

Model 1 – adjusted for patient characteristics: age, sex, race, comorbidities, total number of chronic conditions, and patient payor type

Model 2 – adjusted for Model 1 + physician/practice characteristics: United States Census Region, metropolitan statistical area, solo practice, physician specialty, physician compensation, practice ownership, and physician employment status

^a^CKD and hypertension and nonmissing blood pressure reading

^b^CKD and diabetes and nonmissing HbA1c data

In multivariable analyses adjusted for patient characteristics, the odds of controlled hypertension, control diabetes, ACEi/ARB use, and statin use among patients with CKD was not statistically significantly different across practice reimbursement types. In analyses controlled for both patient and physician/practice characteristics, reimbursement type was also not associated with statistically significant differences in CKD quality indicators. In our sensitivity analyses, capitated reimbursement was not associated with statistically different odds of controlled hypertension or controlled diabetes (HbA1c < 8%) in fully adjusted models (Supplemental Table 2).

## Discussion

In this nationally representative sample of US outpatient visits, we found that about 9% of patients with chronic disease visited practices that receive the majority of their revenue from capitated payments. We also found that practices with majority capitated revenue differed significantly from FFS and other practices in patient characteristics, serving a more diverse patient population and having a reduced visit frequency (1.5 fewer visits in the past year), and in physician and practice characteristics, including their region, physician compensation, practice ownership, and payor mix. Lastly, we found that reimbursement type was not associated with consistent differences in hypertension, diabetes, or CKD quality indicators.

While the associations found in this study do not necessarily indicate causation, we find important differences in chronic disease quality of care by reimbursement type that complements prior literature. A Cochrane systematic review found only 4 studies meeting inclusion criteria with variable study settings, and also found that FFS care was associated with increased visit frequency [13]. Analyses in the 1990s showed that capitation was associated with reduced utilization of laboratory tests and overall charges for patients with hypertension [27], and was not associated with adverse health status in an elderly Medicaid population [28]. On the other hand, a systematic review of 35 studies found that managed care plans, which frequently had capitation payment arrangements, had mixed effects on quality of care, and several studies showed negative effects of managed care on enrollees with chronic conditions [29]. Another systematic review found that FFS care, compared with capitation, was associated with more patient visits and greater continuity; however, the review was limited by study heterogeneity and the low number of eligible studies [13]. More recent evidence from Canada showed that capitation compared with FFS was associated with similar quality of care for diabetes, congestive heart failure, and coronary artery disease [30], but better quality of care for hypertension [31], weight monitoring, and smoking cessation [32]. Contemporary evidence from a population-based primary care system in Hawaii demonstrated that capitated payments were associated with improvements in a Healthcare Effectiveness Data and Information Set (HEDIS) composite quality measure score, as well as a reduction in number of visits [33]. Taken together, evidence from a variety of settings and disease entities does not point to an overall conclusion about the impact of capitated payments on chronic disease quality of care.

Several factors may explain these mixed findings. First, the capitation payment amount should also be considered, which may differ substantially across Medicare, Medicaid, and commercial managed care settings [34]. Second, practices are subject to different quality metrics and pay-for-performance initiatives depending on the payor arranagement, which incentivize quality of care improvements [35, 36]. Thus, the impact of not only capitation vs. FFS reimbursement type, but also quality metrics and other regulatory requirements, impact quality of care delivery and must be considered in evaluating new capitation models [37, 38].

A key strength of our study is that our analysis leveraged a data source with practice revenue information integrated with patient and clinical encounter information. Our results have policy implications for the upcoming new capitation models, PCF and KCC. First, our findings provide a detailed picture of patient and practice characteristics where capitation is currently implemented. Practices already familiar with capitated arranagements may more readily elect into the PCF and KCC models, and have infrastructure in place to support practice changes. Our finding that practices with majority capitation reimbursement served more Black and Hispanic patients likely represents a greater enrollment of these patients in managed Medicaid plans. Given racial and ethnic disparities in chronic disease management, the racial/ethnic composition of majority capitation practices may influence chronic disease care, and we adjusted for this in our analyses [39, 40]. Second, our results provide baseline estimates of quality of care performance by practice reimbursement composition. The outcomes we analyzed align with the hypertension and diabetes quality measures incentivized in PCF, and the hypertension control measure in KCC. Notably, we found that ACEi/ARB use and statin use were suboptimal across practice reimbursement types, and dedicated quality measures for these medications tied to financial incentives should be considered in PCF and KCC.

Future analyses should evaluate the effect of PCF and KCC model implementation on quality and utilization of care, using quasi-experimental research designs such as differences-in-differences methods. Several important considerations for these analyses deserve mention. First, practice reimbursement is separate from physician compensation. Although PCF and KCC practices will be reimbursed with capitated payments, physicians may still be incentivized toward in-person visits if they face relative value unit (RVU) targets in tiered compensation systems. Thus, a key component of PCF and KCC evaluations will be understanding the relationships between capitated reimbursements, physician compensation structures, and care delivery [41]. Second, many population health management strategies incentivized under PCF and KCC, such as panel management or care coordination activities, will not be readily measurable from claims data. Therefore, qualitative and quantitative methodologies will be crucial to evaluate the effectiveness of care delivery changes under PCF and KCC. Third, in addition to capitated payments, PCF and KCC introduce other simultaneous co-interventions, including a focused set of quality metrics, a learning collaborative, and performance incentives, so changes in quality and utilization will not be attributable solely to capitated payments. Lastly, KCC is a Medicare-only model, and lack of participation from other payors may limit care delivery changes [11], particularly in practices with predominantly commercially insured or Medicaid populations.

There are are several limitations to consider in interpreting our results. First, our assessment of hypertension control was limited to a single office BP reading, as home BP readings were not available in the dataset. We did not include more expansive measures of quality of care, such as patient-reported outcomes and healthy behaviors. Second, we identified CKD using ICD-9 and ICD-10 diagnosis codes which are insensitive for CKD [42]. Further, CKD coding sensitivity may differ by practice setting, which could bias our results. Third, our study is observational and does not exploit exogenous variation in reimbursement type, so our results may be more subject to residual confounding. Our results may have had limited power to detect statistically significant differences in chronic disease care by practice reimbursement type. Furthermore, socioeconomic status, education, and employment variables were not available in our data source, which are key determinants of chronic disease care. Also, we included free-standing office-based practices and not hospital-based outpatient practices because the National Hospital Ambulatory Medical Care Survey (NHAMCS) data releases were not available after 2011, which somewhat limits the generalizability of our findings. Lastly, we used reimbursement information at the practice-level, rather than the visit-level, so were unable to know if a particular visit was paid for with capitation or FFS [7].

## Conclusions

In summary, we found that a substantial subset of patients with chronic disease receive care in capitation-based settings. Capitated practices had distinctively different patient and practice characteristics, when compared with FFS practices, but capitated reimbursement was not associated with consistent differences in hypertension, diabetes, and CKD quality indicators. Amidst the COVID-19 pandemic and as the US enters into demonstration projects that introduce capitated payments for primary care and CKD, it will be crucial to rigorously examine the impact of reimbursement changes on chronic disease quality of care.

## Supplementary Information

**Additional file 1.**

## Data Availability

All data are publicly available on the Centers for Disease Control & Prevention website: https://www.cdc.gov/nchs/ahcd/index.htm.

## Abbreviations

CKD: Chronic kidney disease
ACEi: Angiotensin converting enzyme inhibitor
ARB: Angiotensin receptor blocker
NSAID: Nonsteroidal anti-inflammatory drug
HbA1c: Hemoglobin A1c
ICD: International Classification of Diseases
JNC: Joint National Committee
KDOQI: Kidney Disease Outcomes Quality Initiative
ADA: American Diabetes Association
KDIGO: Kidney Disease Improving Global Outcomes
