# Supplementary material for: Capitated versus fee-for-service reimbursement and quality of care for chronic disease: a US cross-sectional analysis
Source: BMC Health Serv Res. 2022 Jan 4;22:19. doi: 10.1186/s12913-021-07313-3 (PMC8723903; doi:10.1186/s12913-021-07313-3)
Supplement: Supplementary file 1 — Additional file 1. [file 12913_2021_7313_MOESM1_ESM.docx]

**Supplemental Table 1. Characteristics Associated with Being a Majority Capitated Practice, Among Visits for Chronic Disease (N = 41,897).**

|  | **Majority Capitation**  **aOR**  **(95% CI)** | **p-value** |
| --- | --- | --- |
| **Patient Characteristics** | | |
| **Demographics** | | |
| Age (per 10 years) | 1.06  (0.96 – 1.16) | 0.230 |
| Sex | | |
| Male | 0.95  (0.77 – 1.17) | 0.630 |
| Female *(ref.)* | 1 | -- |
| Race/Ethnicity | | |
| Non-Hispanic White *(ref.)* | 1 | -- |
| Non-Hispanic Black | 2.17  (1.41 – 3.35) | <0.001 |
| Hispanic | 2.85  (1.51 – 5.35) | 0.001 |
| Non-Hispanic Other | 2.63  (1.39 – 4.97) | 0.003 |
| **Comorbidities** | | |
| Cancer | 0.82  (0.58 – 1.14) | 0.233 |
| Cerebrovascular Disease | 0.76  (0.50 – 1.16) | 0.203 |
| COPD | 1.03  (0.72 – 1.46) | 0.878 |
| Congestive Heart Failure | 1.15  (0.79 – 1.67) | 0.473 |
| Coronary Artery Disease | 1.00  (0.76 – 1.32) | 0.995 |
| Depression | 0.84  (0.61 – 1.15) | 0.271 |
| Obesity | 0.97  (0.77 – 1.21) | 0.771 |
| Total Number of Chronic Conditions (per number increase) | 1.14  (1.03 – 1.26) | 0.015 |
| **Payor Type** | | |
| Private insurance *(ref.)* | 1 | -- |
| Medicare | 1.02  (0.73 – 1.42) | 0.903 |
| Medicaid | 1.37  (0.84 – 2.23) | 0.207 |
| Other | 1.29  (0.70 – 2.38) | 0.422 |
| **Visit Characteristics** | | |
| Number of Times Seen in Past 12 Months (per visit increase) | 0.95  (0.91 – 0.99) | 0.012 |
| **Physician/Practice Characteristics** | | |
| **Geographic Region** | | |
| Northeast *(ref.)* | 1 | -- |
| Midwest | 0.74  (0.33 – 1.67) | 0.465 |
| South | 1.67  (0.72 – 3.87) | 0.234 |
| West | 6.07  (2.91 – 12.64) | <0.001 |
| **Metropolitan Area** | 0.84  (0.44 – 1.60) | 0.586 |
| **Solo Practice** | 0.53  (0.27 – 1.03) | 0.059 |
| **Physician Specialty** | | |
| Primary Care *(ref.)* | 1 | -- |
| Medical Specialty Care | 0.52  (0.28 – 0.95) | 0.034 |
| **Physician Compensation** | | |
| Share of Billings *(ref.)* | 1 | -- |
| Fixed Salary | 3.62  (1.91 – 6.83) | <0.001 |
| Mix | 2.49  (1.25 – 4.93) | 0.009 |
| Other | 2.92  (1.15 – 7.41) | 0.024 |
| **Practice Ownership** | | |
| Physician *(ref.)* | 1 | -- |
| Medical/Academic Health Center | 0.77  (0.35 – 1.69) | 0.509 |
| Insurance Company, Health Plan or HMO | 0.84  (0.44 – 1.60) | 0.599 |
| **Employment Status** | | |
| Full Owner *(ref.)* | 1 | -- |
| Part Owner | 1.30  (0.60 – 2.82) | 0.512 |
| Employee or Contractor | 1.16  (0.53 – 2.53) | 0.704 |
| **Payor Mix** | | |
| Majority Medicare | 2.53  (1.40 – 4.55) | 0.002 |
| Majority Medicaid | 2.90  (0.93 – 9.01) | 0.065 |
| Majority Private Insurance | 2.06  (1.18 – 3.59) | 0.012 |
| Majority Patient Payments or Other* | 1.33  (0.38 – 4.67) | 0.660 |
| **Majority Managed Care Contracts** | 5.68  (3.43 – 9.40) | <0.001 |

Results are from multivariable logistic regression examining the association between characteristics and practices having the majority of revenue from capitation, adjusting for other patient and physician/practice characteristics.

COPD = chronic obstructive pulmonary disease, HMO = Health Maintenance Organization.

*Other includes charity, research, CHAMPUS, and the VA.

**Supplemental Table 2. Results from Sensitivity Analysis of Hypertension and Diabetes Quality Indicators.**

| **Reimbursement Composition** | **Controlled Hypertension (BP<130/80) in Patients with Hypertension^a^**  **% or aOR**  **(95% CI)**  **(N = 36,540)** | **p-value** | **Controlled Diabetes (HbA1c <8%) in Patients with Diabetes^b^**  **% or aOR**  **(95% CI)**  **(N = 6,016)** | **p-value** | **Controlled Hypertension (BP<140/90) in Patients with CKD and Hypertension^a^**  **% or aOR**  **(95% CI)**  **(N = 3,028)** | **p-value** | **Controlled Diabetes (HbA1c <8%) in Patients with CKD and Diabetes^b^**  **% or aOR**  **(95% CI)**  **(N = 579)** | **p-value** |
| --- | --- | --- | --- | --- | --- | --- | --- | --- |
| **Unadjusted Prevalence** | | | | | | | | |
| Majority Capitation | 50% | 0.231 | 77% | 0.547 | 64% | 0.055 | 82% | 0.654 |
| Majority FFS | 54% | *(ref.)* | 79% | *(ref.)* | 77% | *(ref.)* | 79% | *(ref.)* |
| Other Revenue Mix | 53% | 0.633 | 80% | 0.794 | 81% | 0.290 | 84% | 0.528 |
| **Model 1** | | | | | | | | |
| Majority Capitation | 0.86  (0.65 – 1.16) | 0.324 | 0.81  (0.52 – 1.26) | 0.348 | 0.56  (0.34 – 0.91) | 0.019 | 1.30  (0.47 – 3.57) | 0.613 |
| Majority FFS *(ref.)* | 1 | -- | 1 | -- | 1 | -- | 1 | -- |
| Other Revenue Mix | 0.96  (0.80 – 1.16) | 0.696 | 1.23  (0.81 – 1.88) | 0.332 | 1.33  (0.74 – 2.42) | 0.340 | 1.68  (0.47 – 5.99) | 0.419 |
| **Model 2** | | | | | | | | |
| Majority Capitation | 0.86  (0.66 – 1.12) | 0.265 | 0.74  (0.46 – 1.18) | 0.208 | 0.60  (0.37 – 0.98) | 0.042 | 1.52  (0.45 – 5.20) | 0.496 |
| Majority FFS *(ref.)* | 1 | -- | 1 | -- | 1 | -- | 1 | -- |
| Other Revenue Mix | 0.97  (0.80 – 1.16) | 0.732 | 1.15  (0.76 – 1.73) | 0.504 | 1.27  (0.70 – 2.30) | 0.439 | 1.56  (0.58 – 4.25) | 0.377 |

CKD = chronic kidney disease, BP = blood pressure, aOR = adjusted odds ratio, CI = confidence interval, HbA1c = Hemoglobin A1c, ACEi = Angiotensin converting enzyme inhibitor, ARB = angiotensin receptor blocker, NSAID = nonsteroidal anti-inflammatory drug.

Differences in the unadjusted prevalence of quality indicators across reimbursement types were assessed using unadjusted logistic regression.

Model 1 – adjusted for patient characteristics: age, sex, race, comorbidities, total number of chronic conditions, and patient payor type.

Model 2 – adjusted for Model 1 + physician/practice characteristics: geographic region, metropolitan statistical area, solo practice, physician specialty, physician compensation, practice ownership, and physician employment status.

^a^Hypertension and nonmissing blood pressure reading.

^b^Diabetes and nonmissing HbA1c data.
